# The anti-allergic potential of stingless bee honey from different botanical sources via modulation of mast cell degranulation

**DOI:** 10.1186/s12906-023-04129-y

**Published:** 2023-09-04

**Authors:** Poi Yi Aw Yong, Ashley Jia Wen Yip, Fahmida Islam, Hui Jing Hong, Yi En Teh, Chau Ling Tham, Ji Wei Tan

**Affiliations:** 1 School of Science, Monash University Malaysia, Jalan Lagoon Selatan, Bandar Sunway, Subang Jaya, 47500 Selangor Malaysia; 2 Australian Regenerative Medicine Institute, Monash University, Clayton, 3800 VIC Australia; 3 Institute for Research in Molecular Medicine (INFORMM), Universiti Sains Malaysia, Pulau Pinang, 11800 Malaysia; 4 Department of Biomedical Science, Faculty of Medicine and Health Sciences, Universiti Putra Malaysia, Serdang, 43400 Selangor Malaysia; 5 Natural Medicines and Products Research Laboratory, Institute of Bioscience, Universiti Putra Malaysia, Serdang, 43400 Selangor Malaysia

**Keywords:** Stingless bee honey, Kelulut honey, Botanical sources, Anti-allergic, Anti-inflammation, Human mast cells, PMACI, Polyphenols, In silico

## Abstract

**Background:**

Allergy is an inflammatory disorder affecting around 20% of the global population. The adverse effects of current conventional treatments give rise to the increased popularity of using natural food products as complementary and alternative medicine against allergic diseases. Stingless bee honey, commonly known as Kelulut honey (KH) in Malaysia, has been used locally as a traditional remedy to relieve cough and asthma. This study evaluated the anti-allergic potential of KH collected from four different botanical sources on phorbol ester 12-myristate-3-acetate and calcium ionophore-activated human mast cells.

**Methods:**

The present study examined the inhibitory effects of all collected honey on the release of selected inflammatory mediators, such as tumor necrosis factor-α (TNF-α), interleukin (IL)-4, IL-6, IL-8, histamine, and β-hexosaminidase in an activated HMC. Besides that, all honey's total phenolic content (TPC) was also examined, followed by using liquid chromatography with tandem mass spectrometry (LC–MS/MS) to identify the phytochemicals in the honey. Further examination of the identified phytochemicals on their potential interaction with selected signaling molecules in an activated mast cell was conducted using computational methods.

**Results:**

The results indicated that there were significant inhibitory effects on all selected inflammatory mediators’ release by KH sourced from bamboo (BH) and rubber tree (RH) at 0.5% and 1%, but not KH sourced from mango (AH) and noni (EH). BH and RH were found to have higher TPC values and were rich in their phytochemical profiles based on the LC–MS/MS results. Computational studies were employed to determine the possible molecular target of KH through molecular docking using HADDOCK and PRODIGY web servers.

**Conclusions:**

In short, the results indicated that KH possesses anti-allergic effects towards an activated HMC, possibly by targeting downstream MAPKs. However, their anti-allergic effects may vary according to their botanical sources. Nevertheless, the present study has provided insight into the potential application of stingless bee honey as a complementary and alternative medicine to treat various allergic diseases.

**Supplementary Information:**

The online version contains supplementary material available at 10.1186/s12906-023-04129-y.

## Background

Allergic diseases also known as Type I hypersensitivity reactions are one of the most prevalent diseases in the modern world. It is characterized by an aberrant response of the human body towards low levels of environmental allergens that are otherwise ubiquitous [1]. This unfavorable immune response initiates inflammatory symptoms in various organs, leading to a plethora of allergic diseases such as food allergy, atopic dermatitis, allergic asthma or allergic rhinitis [2]. In general, allergic diseases are affecting 10 to 20% of adults and children in developed countries. Allergic disease symptoms may vary according to their type. However, some symptoms such as difficulties in breathing, rashes, nausea, and diarrhea are more commonly seen in allergic patients [3, 4]. This heightened the concern to find effective solutions in combating this global health threat.

Even though there are various allergic diseases, all of them share several common characteristics in terms of their inflammatory processes. Studies have reported that mast cell (MC) is one of the main key players that trigger an allergic reaction through its degranulation process [5, 6]. The underlying mechanisms in MC degranulation are dependent on the calcium signalling pathway, in which calcium ions will act as a second messenger during the activation of MC. Hence, an essential trigger of MC activation is reliant on the influx of calcium ions into the cell [7]. The use of synthetic compounds such as phorbol 12-myristate 13-acetate (PMA) and calcium ionophore (CI) would increase the intracellular calcium ions concentration in vitro models of MC degranulation. This has been demonstrated in cell lines such as the Human Mast Cell line (HMC-1), which are immature transformed cell lines that do not express functional IgE receptors [8, 9]. Therefore, albeit there is a lack of functional IgE expression in HMC-1, this cell line still permits the expression of various mediators such as histamine and cytokines [9], allowing it to replicate MC degranulation in an in vitro model, which permits experimentation of various treatment options for allergic reactions.

Among the plethora of allergic disease management, antihistamines are one of the most common prescriptions to relieve allergic symptoms [10]. Currently, there are three generations of antihistamines. While effective in its treatment, strong sedative effects have been observed in first-generation antihistamines due to their ability to penetrate easily through the blood–brain barrier causing drowsiness in patients. Furthermore, an overdose of second and third-generation antihistamines has been observed to inflict fatigue, headache, and drowsiness. These adverse effects negatively impacted the patient’s quality of life [11]. Apart from antihistamines, other approaches such as allergen-specific immunotherapy are employed in the treatment of allergies which expose a small number of allergens to patients in hopes of improving the tolerance of the patient towards it [12]. This form of therapy involves subcutaneous administration of gradually increasing quantities of a patient’s corresponding allergen until an ideal dose capable of stimulating immune tolerance toward the allergen is achieved [12]. However, there is a chance to trigger local and systemic reactions such as redness and itching or even anaphylaxis [13]. Thus, the adverse effects observed in current treatments of allergic diseases highlight the significance of exploring alternative methods to combat the disease.

In the recent decade, science has been able to prove that natural food products rich in components such as flavonoids and phenols are potentially eliciting anti-inflammatory properties observed in allergies with a prospect of lesser side effects or idiosyncratic reactions. This underpins the need for further investigation into natural products and their anti-inflammatory potential to manage allergies. Honey is a natural food product produced by bees from liquid plant exudates. In the past, honey was often employed for wounds, coughs, colds, and respiratory diseases [14]. In recent times, scientific evidence has proven that honey possesses valuable bioactivities such as antibacterial, anti-allergy, antioxidant, anticancer, antidiabetic, and wound healing. However, most of these studies focused on honey produced by *Apis mellifera* bees, with limited studies on stingless bee honey (SBH) due to the smaller distribution of stingless bees [15] and their lower honey production [16]. Stingless bees are mainly distributed in tropical and subtropical regions where they can be classified into two genera, *Melipona* and *Trigona* [15]. SBH, also known locally as Kelulut honey (KH) in Malaysia, is composed mainly of carbohydrates, amino acids, water, ash, proteins, phenolic compounds, and minerals [17]. Among these components, the diverse phenolic and flavonoid compounds play a significant role in their bioactivities. Recent studies have demonstrated that the distinctive phenolic and flavonoid compounds found in SBH exhibited significant anti-inflammatory, antioxidant, and antimicrobial activities [16, 18]. Interestingly, many studies have reported that SBH contains a higher level of bioactive components than *A. mellifera* honey which subsequently leads to higher biological activities [19]. On top of that, botanical sources and geographical regions are known to contribute greatly to the phytochemical properties along with the therapeutic properties of honey [20]. Taken together, all these reported studies show SBH is a strong candidate for further investigation in its beneficial properties as it holds a promising prospect in contributing to the management of allergic diseases. Thereby, the present study aims to investigate the anti-allergy activities of Malaysia KH from four different botanical sources: *Bambusa* (bamboo), *Hevea brailiensis* (rubber), *Mangifera indica* (mango), and *Morinda citrifolia* (noni) plants.

## Materials

### Chemical and reagents

The Isocove’s Modified Dulbecco’s Medium (IMDM) (17,633), α-thioglycerol (M6145), phorbol 12-mysirate 13-acetate (PMA) (P8139), calcium ionophore A23187 mixed calcium magnesium salt (CI) (C4403), p-nitrophenyl *N*-acetyl-β-D-glucosamine (PNAG) (N9376), cromolyn sodium salt (C0399), Folin-Ciocalteu’s phenol reagent (F9252) were purchased from Sigma-Aldrich Corporation (St. Louis, Missouri, USA). Fetal bovine serum (FBS) (FBSEU500) was obtained from Tico Europe (Amstelveen, EU). Penicillin–streptomycin mixed solution (09267–34) and Bovine Serum Albumin (BSA) (01860–07) were purchased from Nacalai Tesque, Inc. (Kyoto, Japan). Dimethyl sulfoxide (DMSO) was purchased from VWR Life Science (Radnor, Pennsylvania, USA). Cell counting kit-8 (CCK-8) reagent (D025-30) was purchased from Bridgen (Beijing, China). Enzyme-linked Immunosorbent Assay (ELISA) kits for TNF-α (DY210), IL-6 (DY206), IL-8 (DY208), and IL-4 (DY204-5) were purchased from R&D Systems, Inc. (Minnesota, USA), while histamine ELISA kit (NBP2-62,860) was purchased from Novus Biologicals, LLC (Centennial, Colorado, USA). Pierce™ 3,3’,5,5’tetramethylbenzidine (TMB) Substrate Kit (PI34021) was purchased from Thermo Scientific™ (Rockford, Illinois, USA).

### Cell culture

Human mast cells (HMC-1) (SCC062) were purchased from Merck Millipore Corporation (Burlington, Massachusetts, USA). Cells were maintained in IMDM supplemented with 10% FBS, 1.2 mM α-thioglycerol and antibiotics (100 U/mL penicillin and 100 µg/mL streptomycin) at 37℃ with 5% CO_2_ humidified incubator. The cells within passages 5 to 11 were used for experiments throughout this study.

### KH samples

The KH samples were obtained from DinoKelulut bee farm located in Negeri Sembilan, Malaysia. The raw honeys were collected from the matured propolis pot of stingless bee species, *Trigona Itama*, using a sterile Pasteur pipette. Four types of monofloral KH were collected and named accordingly based on the botanical sources, i.e. *M. indica* (AH), *Bambusa* (BH), *M. citrifolia* (EH), and *H. brasiliensis* (RH). Monofloral honey is produced by a single botanical source containing mainly its nectar with minor nectar contribution from other plants. Monofloral honey is produced by a single botanical source containing mainly its nectar with minor nectar contribution from other plants [21]. All raw honeys were stored at 4 °C. KHs were diluted with incomplete IMDM to 10% stock concentration and adjusted to pH 6.9—7.1 before being sterilised with a 0.22 µm polyethersulfone membrane syringe filter.

## Methods

### CCK cell viability assay

The cytotoxicity of the four different types of KH against HMC-1 cells were determined using a CCK cell viability assay kit. The assay was performed according to the manufacturer’s instructions. Briefly, HMC-1 cells (1 × 10^5^ cells/well) were seeded into 96-well plates and incubated overnight. The seeded cells were then treated with various concentrations of honey ranging from 0.01% to 5% (w/v) followed by another 24 h incubation. After the incubation period, 10 μL of CCK reagent was added to each well. After 2 h of incubation, the absorbance (OD) was measured at 450 nm. The cell viability was calculated using the following formula:

$$\mathrm{Cell\ viability }\left(\mathrm{\%}\right)=\frac{\mathrm{Mean\ OD\ of\ treatment\ groups}}{\mathrm{Mean\ OD\ of\ normal\ control\ group}}\times 100\%$$

Three non-cytotoxic concentrations of KH were chosen from the cell viability assay and used in subsequent experiments.

### β-hexosaminidase and histamine release assay

To determine the effects of KH on the release of preformed mediators during MC degranulation, the release of β-hexosaminidase and histamine upon treatment with KH was examined. HMC-1 cells (8 × 10^5^ cells/well) were seeded into 24-well plates and incubated overnight. The cells were treated with three non-cytotoxic concentrations of KH (0.25%, 0.5%, and 1%) cell viability assay for 1 h followed by 2 h induction with 20 nM of PMA and 1 µM of CI (PMACI) at 37℃ in a 5% CO_2_ humidified incubator. The level of histamine in the culture supernatant was measured by using a Histamine EIA kit according to the manufacturer’s instructions.

The measurement of β-hexosaminidase release activity was carried out by following previous published protocol with slight modification [22]. Briefly, the collected culture medium was centrifuged (300 × g, 3 min), and the supernatant (50 μL) was transferred into separate wells. Triton X-100 (1%) were added to wells containing cell lysate for 5 min to release intracellular β-hexosaminidase. Substrate solution (1 mM p-nitrophenyl N-acetyl-β-D-glucosamine in 0.2 M citrate buffer, pH 4.5) was added to both cell supernatant and lysate prior to 1 h incubation. The reaction was then terminated with the addition of a stop solution (0.1 M glycine, pH 10) followed by the transfer of both cell supernatant and lysate (200 μL) into non-treated 96-well plates where the absorbance was measured at 405 nm. The release of β-hexosaminidase was calculated using the following formula:

$$\mathrm{Release\ of\ \beta }-\mathrm{hexosaminidase }\left(\mathrm{\%}\right)= \frac{\mathrm{OD\ supernatant}}{\mathrm{OD\ supernatant }\ +\mathrm{ OD\ lysate}} \times 100\%$$

### Proinflammatory cytokine ELISA assay

To determine the effects of KH on the release of de novo mediators, HMC-1 cells (1 × 10^5^ cells/well) were seeded into 96-well plates and incubated overnight. The cells were then pre-treated with three non-cytotoxic concentrations of KH (0.25%, 0.5%, and 1%) for 1 h, followed by 24 h induction with PMACI at 37℃ in a 5% CO_2_ humidified incubator. The culture medium was centrifuged (300 × g, 2.5 min), and the supernatant collected was stored at—20 °C until further use. The levels of IL-4, IL-6, IL-8, and TNF-α released were measured using respective ELISA assay kits according to the manufacturer’s protocol.

### Total phenolic content (TPC)

The TPC of KHs was determined using the TPC assay as described by Chai et al. (2021) with slight modification [23]. In brief, 0.1 g of KH was diluted with 1 mL of ultrapure water. Diluted KH (0.2 mL) was mixed with 7.5% (w/v) sodium carbonate (Na_2_CO_3_) solution (0.8 mL) and 10% (v/v) Folin-Ciocalteu reagent (1 mL). The mixtures were incubated in the dark for 30 min at room temperature before measuring the absorbance at 765 nm using Spark® Multimode Microplate Reader, Tecan (Männedorf, Switzerland). The TPC results were the mean value of triplicate data expressed as mg of gallic acid equivalent (GAE) per 1 kg of KH.

### Liquid Chromatography with Tandem Mass Spectrometry (LC–MS/MS) analysis

To identify the polyphenol content of KHs used in this present study, all four KHs were analyzed using LC–MS/MS analysis. Briefly, KHs were diluted with ultrapure water in a 1:1 ratio and filtered with a 0.22 µm Polyethersulfone membrane filter before being subjected to LC–MS/MS analysis. The small molecule content of KHs was identified using Agilent 6520 Accurate Mass Q-TOF LC/MS equipped with Dual ESI as the ionization source. Agilent Eclipse XDB-C18 Narrow bore (50 mm × 2.1 mm, 3.5-micron (P/N 930990–902)) was used for analysis. The column temperature was set at 25 °C while the autosampler temperature was set at 4 °C. The flow rate of LC was maintained at 0.5 mL/min. The solvent system included Solvent A (0.1% formic acid in water) and Solvent B (0.1% formic acid in acetonitrile). The data collected were compared with the Metlin database and literature to identify the polyphenols. The relative amount of each polyphenol was identified based on the area under the curve for respective peaks, using the equation as follows:

$$\mathrm{Relative\ quantity\ of\ polyphenol }\left(\mathrm{a}.\mathrm{u}.\right)= \frac{1}{2} \times \mathrm{Height\ of\ peak }\times \mathrm{Width\ of\ peak}$$

### Protein–ligand binding interaction analysis

#### Phytochemical retrieval

Molecular docking approach was employed for binding interaction analysis between the identified polyphenol compounds and selected cellular signalling protein molecules. Briefly, the 2D structure of the polyphenol compounds of KH were obtained from the PubChem database (https://pubchem.ncbi.nlm.nih.gov/). They were subjected to the LigPrep module of Schrodinger Maestro v10.1, and converted to the three-dimensional (3D) structure by including ionization, variation, stereochemical, correction, and energy minimization and optimization of geometry. The optimized polyphenol ligands were then converted to Protein Data Bank format (.pdb) format for use in molecular docking.

#### Selection and preparation of target signalling protein molecules

The 3D structures of targeted signalling protein molecules, namely p38α (PDB ID: 6HWV), ERK (PDB ID: 4QTB), JNK (PDB ID: 3ELJ), NF-κB (PDB ID: 1NFI), PKC alpha (PDB ID: 4RA4), and Calmodulin-1 (PDB ID: 6M7H) were retrieved from RCSB Protein Data Bank (RCSB PDB) (https://www.rcsb.org/) database. Python molecule (PyMOL) viewer software (http://www.pymol.org) was used to refine these protein structures by removing the ligand, assigning bond orders, adding missing hydrogen atoms and disulphide bonds, as well as removing water molecules. The active site prediction in targeted proteins was carried out using SPPIDER II (http://sppider.cchmc.org/).

#### Molecular docking

The molecular docking between targeted signalling protein molecules and polyphenol ligands was carried out using the HADDOCK server [24, 25]. The data were collected, and the best docking models of each signalling protein were determined based on HADDOCK scores. The binding affinity of the protein–ligand complex was also determined using protein binding energy prediction server (PRODIGY) (https://wenmr.science.uu.nl/prodigy/) [26]. The interactions between the proteins and ligands from the best-docked complexes were identified using Protein–Ligand Interaction Profiler (PLIP) web server (https://plip-tool.biotec.tu-dresden.de/plip-web/plip/index) [27]. The results were then visualized using PyMol (http://www.pymol.org; DeLano Scientific, San Carlos, California, USA).

### Statistical analysis

All assays described, except for the computational study, were conducted in three technical replicates. All results (including the molecular docking) generated were expressed as mean ± standard error of mean (SEM) from three repeated independent experiments (*n* = 3). Statistical analyses were performed using SPSS V21 (Chicago, IL, USA). One-way analysis of variance (ANOVA) followed by Tukey's test was used to compare the results of different treatment groups with the normal control group (black bar). A *P* value of less than 0.05 is considered statistically significant. Unless otherwise stated, all statistical analyses were performed as mentioned.

## Results

### Cytotoxic effect of KH on HMC-1 cells

**Fig. 1:**
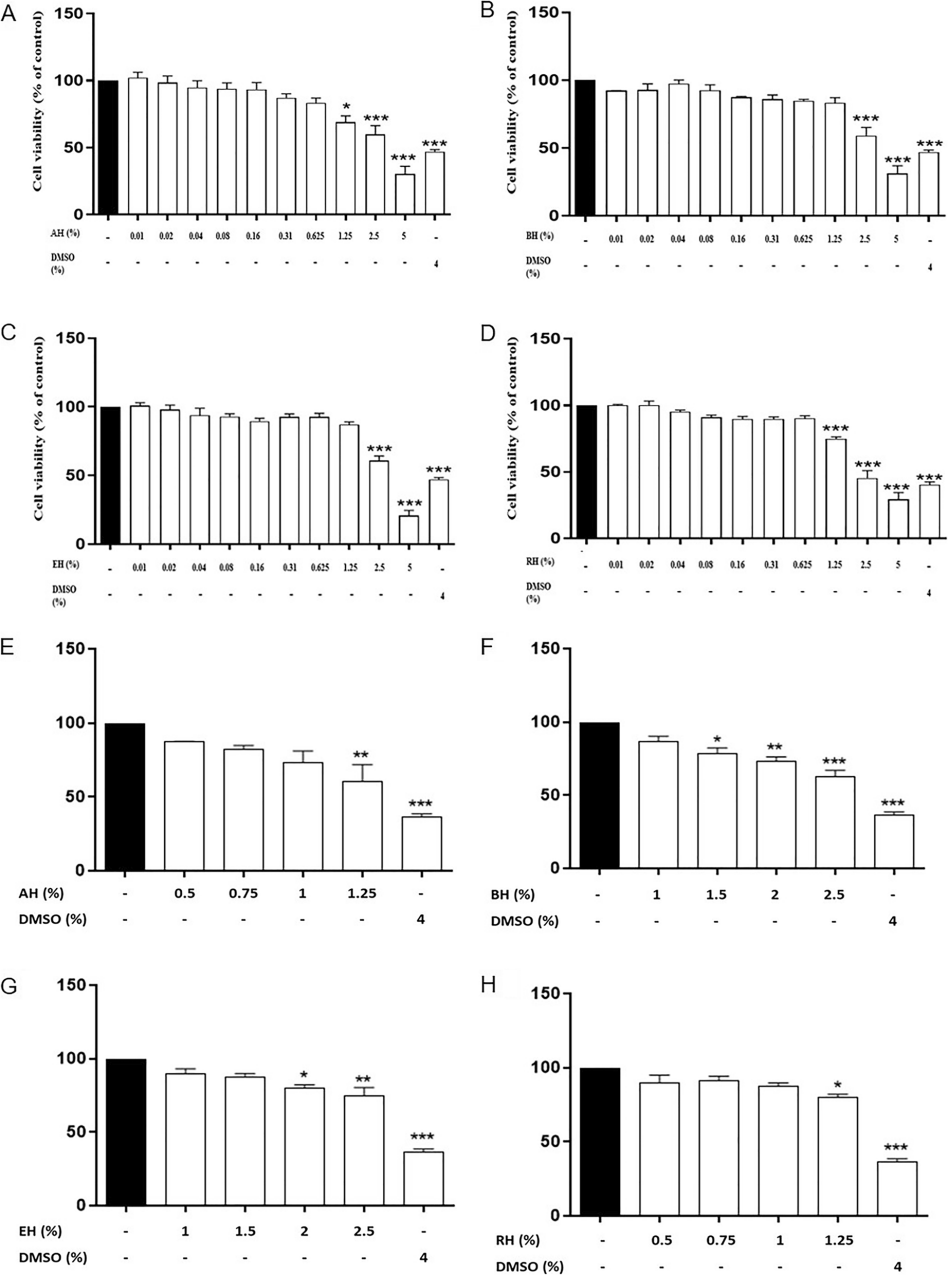
Cytotoxic effect of Kelulut honey on HMC-1 cells. The PMACI-induced cells were incubated without (normal control) or with increasing concentrations of Kelulut honey (0.01 – 5%) for 24 h. The DMSO treatment group was used as a positive control. The cytotoxicity of the treatments AH (**A** and **E**), BH (**B** and **F**), EH (**C** and **G**), and RH (**D** and **H**) were determined using a CCK cell viability assay. The results are expressed as mean ± SEM values of three repeated independent experiments. *P < 0.05 and *** *P* < 0.005 as compared to the normal control group (black bars)

To ensure that the inhibitory effects of KHs on the degranulation of MC were not from cytotoxicity on HMC-1 cells, the cytotoxic effects of AH, BH, EH, and RH on HMC-1 cells were determined using CCK-8 cell viability assay. This assay is based on the cellular dehydrogenase activity which converts WST-8 (2-(2-methoxy-4-nitrophenyl)-3-(4-nitrophenyl)-5-(2,4-disulfophenyl)-2H-tetrazolium, monosodium salt) into a water-soluble formazan [28]. Figure 1B and C indicate that BH and EH significantly reduced the cell viability of HMC-1 cells at the concentration of 2.5% and above (*P* < 0.005). On the other hand, RH and AH significantly reduced the cell viability of HMC-1 cells at the concentration of 1.25% and above (Fig. 1A and D) (*P* < 0.05). A follow-up cytotoxicity screening (Fig. 1E to H) shows that all four KH do not show any significant cell viability drop at 1% honey treatment. Thus, three non-cytotoxicity concentrations (0.25, 0.5, 1%) of KHs were selected for subsequent experiments in this current study.

### Effect of KH on the level of pre-formed mediators released

**Fig. 2:**
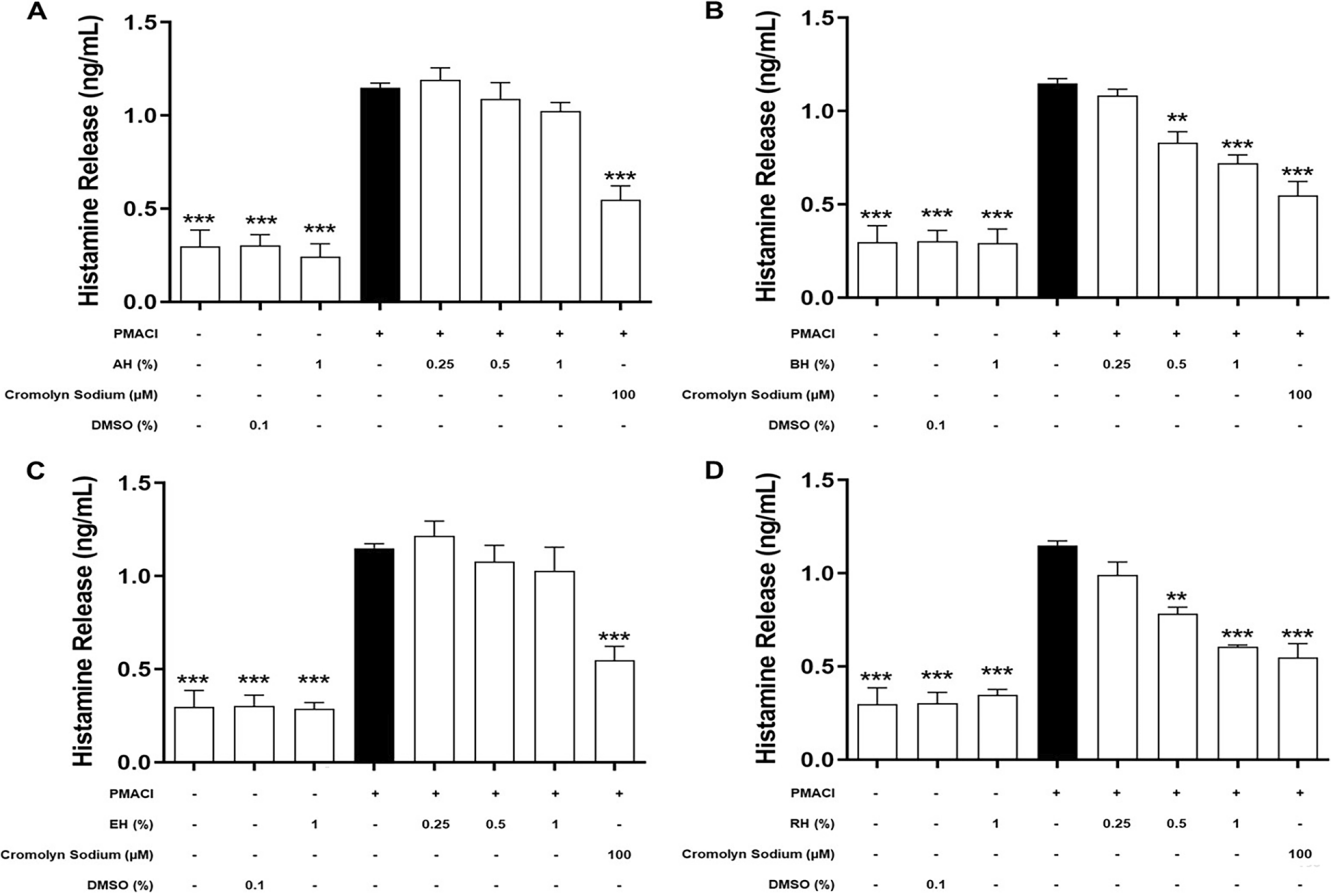
The effects of (**A**) AH, (**B**) BH, (**C**) EH, and (**D**) RH on the levels of histamine released by PMACI-induced HMC-1 cells. PMACI-induced cells were pre-treated without (normal control) or with increasing concentrations of KHs (0.25, 0.5, and 1%) for 1 h followed by 24 h induction with PMACI. The cromolyn sodium treatment group was used as a positive control. The level of cytokines released were determined using respective ELISA kits according to the manufacturer’s protocol. Results obtained from three repeated independent experiments are expressed as mean $\pm$ standard error of mean (SEM). ** P < 0.01 and *** *P* < 0.005 as compared to the normal control group (black bars)

**Fig. 3:**
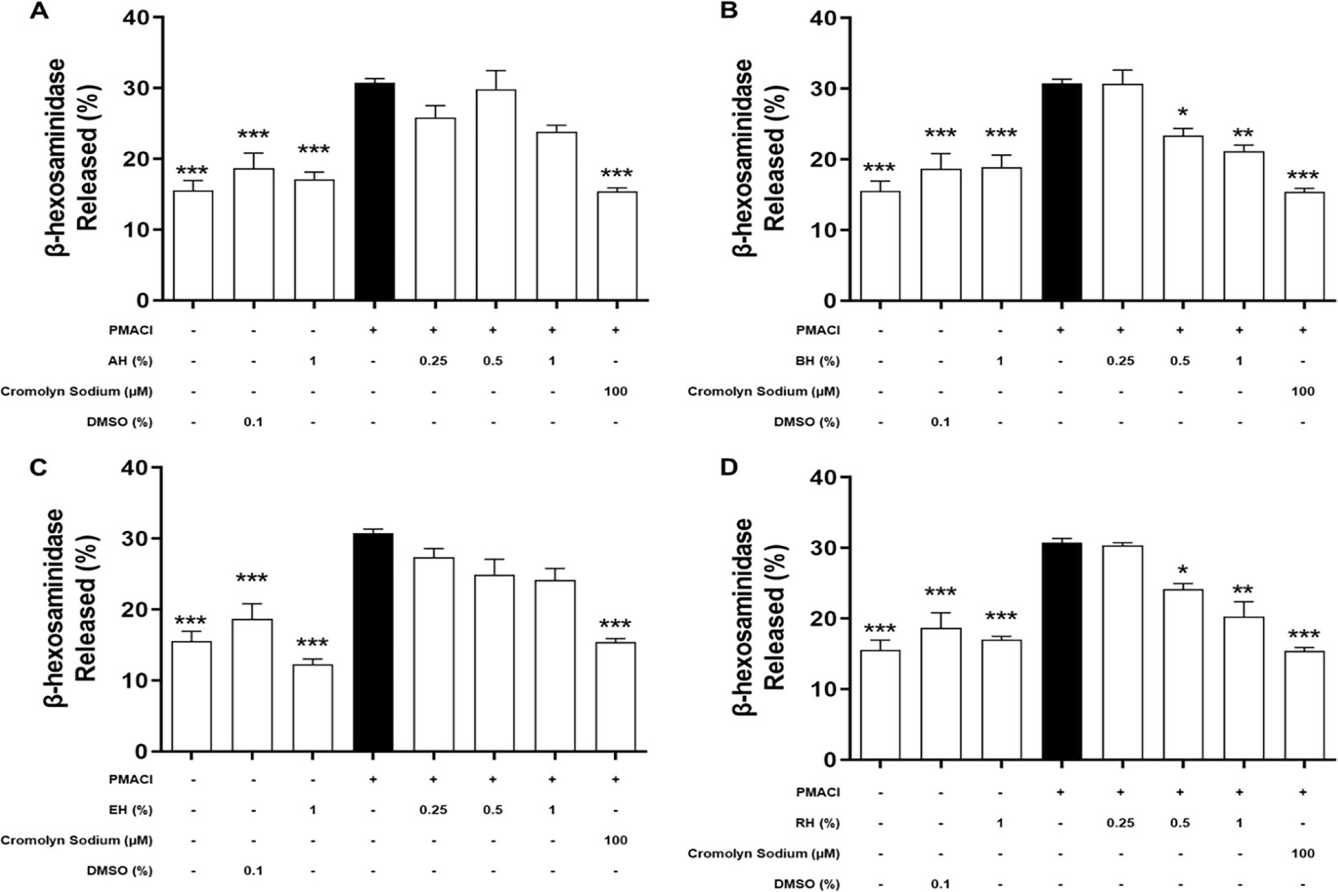
The effect of KH pre-treatment on HMC-1 with (**A**) AH, (**B**) BH, (**C**) EH, and (**D**) RH on the level of β-hexosaminidase released. HMC-1 cells were pre-treated without (normal control) or with respective KH for 1 h and followed by 2 h induction with PMACI. The cromolyn sodium treatment group was used as a positive control. β-hexosaminidase released was quantified by following previous published protocol. Results obtained from three repeated independent experiments are expressed as mean $\pm$ standard error of mean (SEM). * *P* < 0.05, ** *P* < 0.01, *** *P* < 0.005 as compared to the induced control group (black bars)

Pre-formed mediators released by HMC-1 were quantified in order to evaluate the degree of degranulation as histamine (Fig. 2) and β-hexosaminidase (Fig. 3) are the important markers of mast cell degranulation. According to Fig. 2, the induction by PMACI (black bars) significantly increased the release of histamine as much as 3.8-fold (1.15 ± 0.042 ng/mL) in comparison to normal cells (0.30 ± 0.153 ng/mL) (*P* < 0.005). Histamine release was significantly reduced by 37.3% and 43.0% when the cells were pre-treated with 0.5% BH and RH, respectively (*P* < 0.01). A further significant decrease in histamine release was shown in 1% BH and RH pre-treatment groups respectively (50.3% and 63.8%) (*P* < 0.005). However, no significant inhibitory effect was found in 0.25% BH and RH pre-treated HMC-1 cells. Cromolyn sodium was used as a positive drug control in this study. As for the cromolyn sodium pre-treatment group, a 70.8% significant reduction in histamine release was observed (*P* < 0.005). On the other hand, the release of histamine in all the AH and EH pre-treatment groups exhibited no significant difference as compared to the PMACI-induced group.

According to Fig. 3, the PMACI-induced group released 30.8 ± 1.02% of β-hexosaminidase, which was significantly one-fold higher than the normal cells (*P* < 0.005). On the other hand, only KH pre-treatments using BH and RH were shown to exhibit inhibitory effects on the release of β-hexosaminidase. Pre-treatment with BH and RH at 0.5%, respectively, showed a significant 48.6% and 43.5% reduction in the β-hexosaminidase release as compared to the induced group (*P* < 0.05). As the concentration increased to 1%, the β-hexosaminidase release by HMC-1 was significantly inhibited by 67.0% and 68.9% for BH and RH pre-treatment respectively (*P* < 0.01). Release of β-hexosaminidase was also significantly decreased in the cromolyn sodium pre-treatment group, which was approximately 100% reduction in comparison to the PMACI-induced group (*P* < 0.005). In contrast, no significant inhibitory effect was shown in AH and EH pre-treatment at all concentrations, indicating that these two KHs were not effectively inhibiting the degranulation of HMC-1 cells.

### Effect of KH on the level of de novo mediators released

**Fig. 4:**
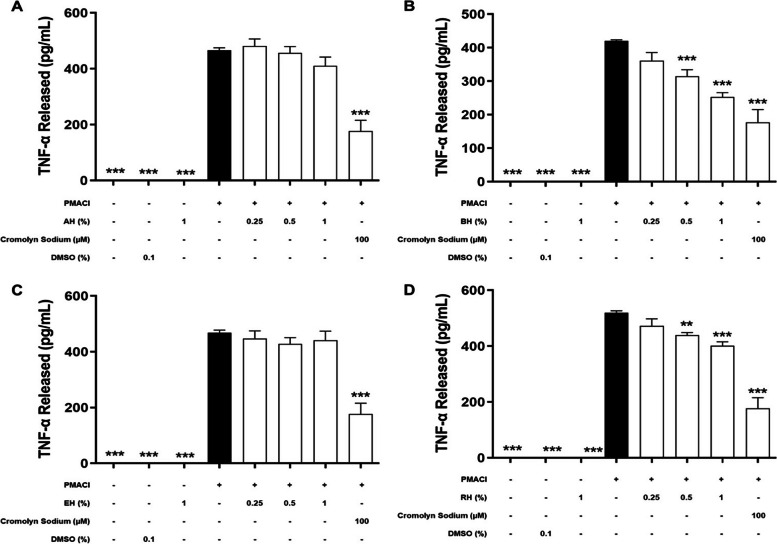
The effects of (**A**) BH, (**B**) RH, (**C**) MH, and (**D**) EH on the levels of TNF-α released by PMACI-induced HMC-1 cells. PMACI-induced cells were pre-treated without (normal control) or with increasing concentrations of honey (0.25, 0.5, and 1%) for 1 h followed by a 24 h induction with PMACI. The cromolyn sodium treatment group was used as a positive control. The level of TNF-α released was determined using an ELISA kit according to the manufacturer’s protocol. Results obtained from three repeated independent experiments are expressed as mean $\pm$ standard error of mean (SEM). ** P < 0.01 and *** *P* < 0.005 as compared to the normal control group (black bars)

**Fig. 5:**
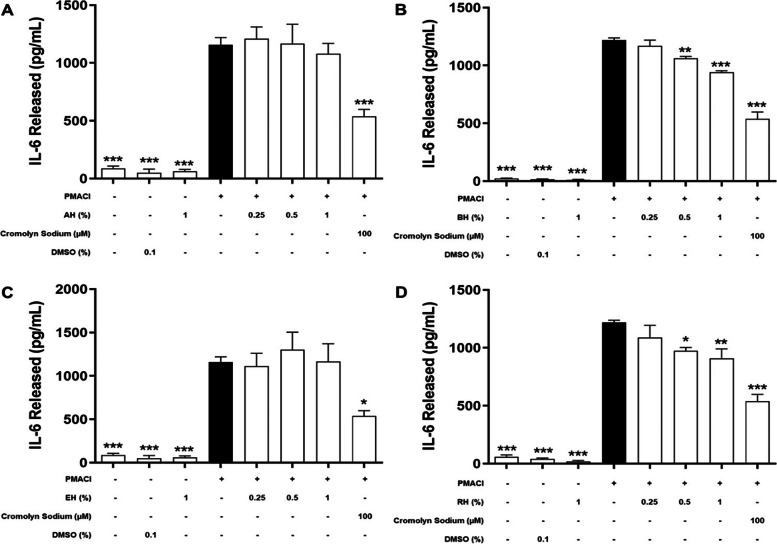
The effects of (**A**) AH, (**B**) BH, (**C**) EH, and (**D**) RH on the levels of IL-6 released by PMACI-induced HMC-1 cells. PMACI-induced cells were pre-treated without (normal control) or with increasing concentrations of honey (0.25, 0.5, and 1%) for 1 h followed by a 24 h induction with PMACI. The cromolyn sodium treatment group was used as a positive control. The level of IL-6 released was determined using respective ELISA kit according to the manufacturer’s protocol. Results obtained from three repeated independent experiments are expressed as mean $\pm$ standard error of mean (SEM). *P < 0.05, ** P < 0.01 and *** *P* < 0.005 as compared to the normal control group (black bars)

**Fig. 6:**
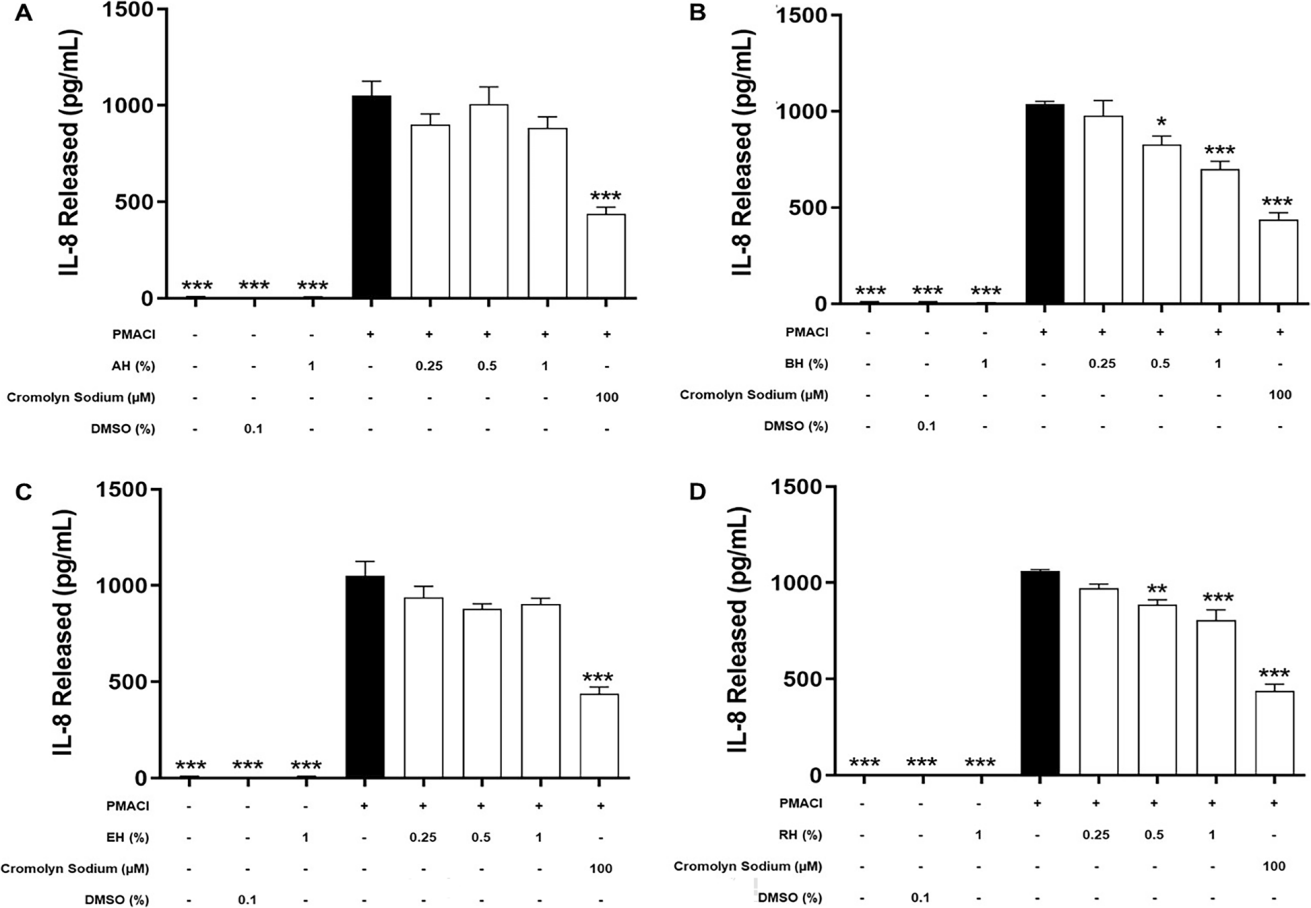
The effects of (**A**) AH, (**B**) BH, (**C**) EH, and (**D**) RH on the levels of IL-8 released by PMACI-induced HMC-1 cells. PMACI-induced cells were pre-treated without (normal control) or with increasing concentrations of honey (0.25, 0.5, and 1%) for 1 h followed by a 24 h induction with PMACI. The cromolyn sodium treatment group was used as a positive control. The level of IL-8 released was determined using an ELISA kit according to the manufacturer’s protocol. Results obtained from three repeated independent experiments are expressed as mean $\pm$ standard error of mean (SEM). *P < 0.05, ** P < 0.01 and *** *P* < 0.005 as compared to the normal control group (black bars)

**Fig. 7:**
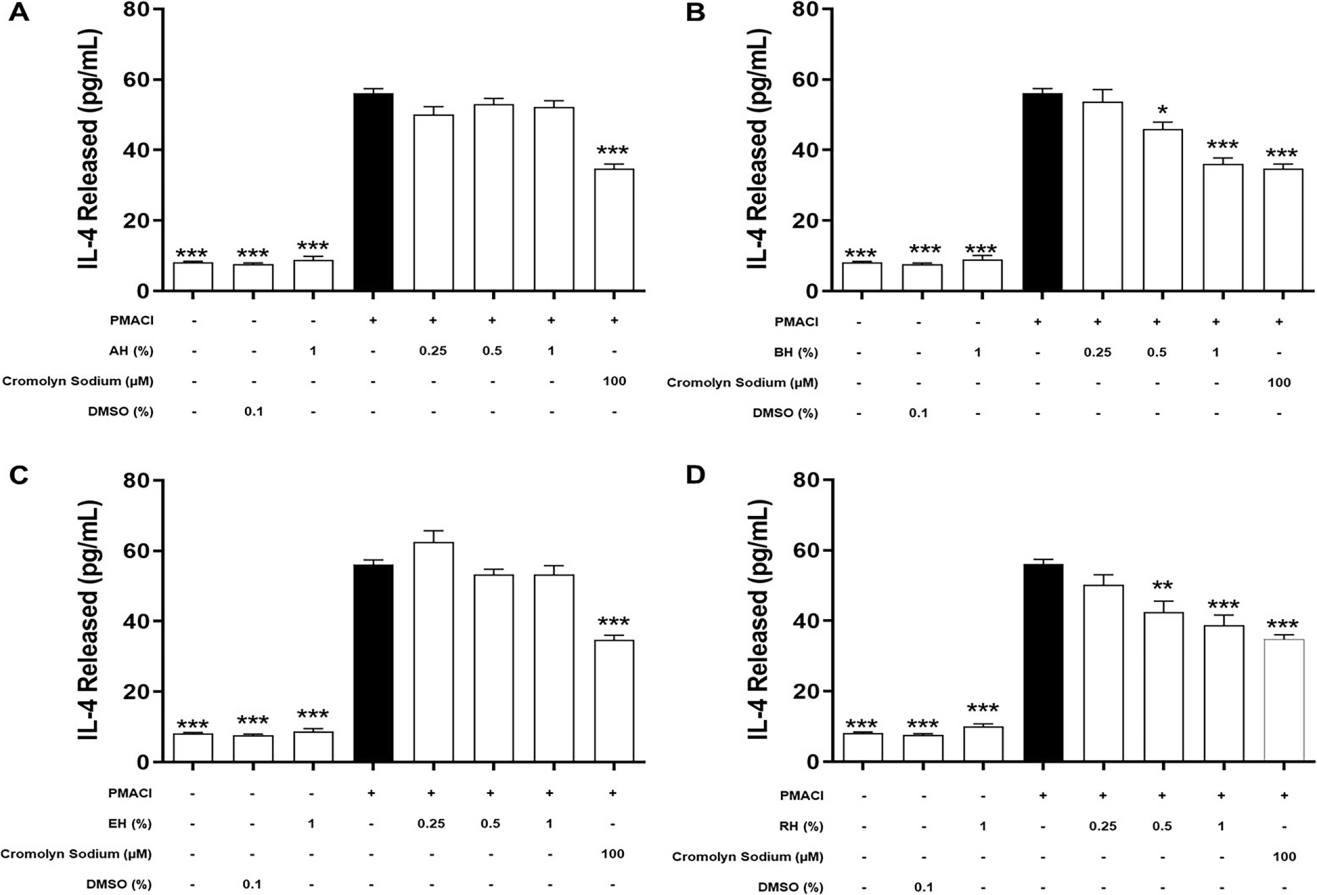
The effects of (**A**) AH, (**B**) BH, (**C**) EH, and (**D**) RH on the levels of IL-4 released by PMACI-induced HMC-1 cells. PMACI-induced cells were pre-treated without (normal control) or with increasing concentrations of honey (0.25, 0.5, and 1%) for 1 h followed by a 24 h induction with PMACI. The cromolyn sodium treatment group was used as a positive control. The level of IL-4 released was determined using ELISA kits according to the manufacturer’s protocol. Results obtained from three repeated independent experiments are expressed as mean $\pm$ standard error of mean (SEM). *P < 0.05, ** P < 0.01 and *** *P* < 0.005 as compared to the normal control group (black bars)

During the late phase of mast cell activation, de novo synthesis inflammatory mediators will be released by HMC-1 cells into the extracellular environment (Amin, 2012). In order to determine the inhibitory effect on de novo synthesized inflammatory mediators, including TNF-α (Fig. 4), IL-6 (Fig. 5), IL-8 (Fig. 6), and IL-4 (Fig. 7) released during an allergic reaction, the HMC-1 cells were pre-treated with KHs for 1 h and followed by a 24 h of induction with PMACI. As shown in all four figures, PMACI induction was able to significantly increase the release of selected de novo mediators by approximately 420 – 520-fold for TNF-α, 13 – 57-fold for IL-6, 157 – 168-fold for IL-8, and 6.9-fold for IL-4 (*P* < 0.005). However, the levels of all de novo mediators released by PMACI-induced HMC-1 cells were significantly reduced in the presence of BH and RH. Pre-treatment of 0.5% and 1% of BH significantly reduced the release of all selected de novo mediators by approximately 13.3%-25.1% and 23.2%-39.9%, respectively in comparison to the PMACI-induced HMC-1 cells (*P* < 0.05). On the other hand, for RH pre-treatment, 0.5% and 1% honey significantly reduced the release of all selected de novo mediators by approximately 15.5%-24.3% and 22.8%-36.2%, respectively in comparison to the PMACI-induced HMC-1 cells (*P* < 0.05). However, no significant inhibition was observed at 0.25% of for both BH and RH on all the de novo mediators tested. Finally, the release of all de novo mediators were also significantly decreased in the cromolyn sodium pre-treatment group, which was approximately 44.6%-58.9% reduction in comparison to the PMACI-induced group (*P* < 0.005).

Contrastingly, pre-treatment of varying concentrations of MH and EH does not show a profound effect in suppressing mast cell degranulation. Based on the observation from Figs. 4, 5, 6 and 7, there is no statistically significant effect on the inhibition of the release of all de novo mediators tested.

### Quantification of total phenolic content of KH

The total phenolic content in all four KHs was quantified using the standard curve of gallic acid and presented in Table 1. All the total phenolic content in KHs was significantly different from each other (*P* < 0.005). The highest value of 2257.95 ± 25.12 mg GAE/kg was found in RH, followed by BH (1015.71 ± 5.58 mg GAE/kg) and EH (909.36 ± 3.64 mg GAE/kg). The KH with the lowest phenolic content is AH, with a value of 665.13 ± 4.42 mg GAE/kg.

**Table 1:** Total phenolic content in Kelulut honey

Samples	Total Phenolic Content (mg GAE/kg)
RH	2257.95 ± 25.12^a^
BH	1015.71 ± 5.58^b^
AH	665.13 ± 4.42^d^
EH	909.36 ± 3.64^c^

^a, b, c, d^The data were expressed as mean ± SEM from three independent experiments. Means with different superscript letters were significantly different at the level of p < 0.005 (as compared to all other KHs). GAE, Gallic Acid Equivalent

### Identification of polyphenol compounds in KH samples

In order to determine the polyphenol profiles of all the KHs in this present study, LC–MS/MS was performed, and the data collected were compared to our standard library information (e.g. retention times, m/z value, and molecular mass). According to Table 2, RH was reported to contain 41 compounds which was the highest number of identified compounds among all KHs. The polyphenol content found in RH was also the highest, which was 15 compounds in total, roughly occupying 36.5% of the total number of compounds. Interestingly, both AH and EH have a greater number of total compounds identified than BH but a lesser number of polyphenols. The percentage of total polyphenols in AH and EH was only 16.7% and 16% respectively. In contrast, 42.9% of the total compounds identified in BH were found to be polyphenols, even though it had the fewest total compounds identified.

**Table 2:** The total number of phytochemicals and polyphenols identified in Kelulut honey

Samples	Total Compounds Identified	Total Polyphenols Identified
AH	18	3
BH	14	6
EH	25	4
RH	41	15

The identified polyphenols’ retention time and their identity are shown in Table 3. According to the table, there are a total number of 16 polyphenols identified in all KHs. Among the identified polyphenols, quercetin-3-*O*-(2''-*O*-alpha-rhamnosyl-6''-*O*-malonyl)-beta-D-glucoside (quercetin glucoside) was the only polyphenol that presented in all four KHs. The relative quantification of each polyphenol was determined based on the area under the curve in the chromatogram and was shown in Table 4. The total relative quantification values of polyphenols for AH, BH, EH, and RH were 3.59, 20.35, 10.88, and 45.84 a.u respectively. Notably, caffeoyl-D-glucose was much higher than other polyphenols with a relative quantification value of 13.51 a.u. in BH and 13.69 a.u. in RH, which greatly increase the total relative amount of polyphenols presented in BH and RH.

**Table 3:** Polyphenols identified in Kelulut honey by LC–MS/MS

Assigned No	Retention Time (min)	Molecular Formula	Polyphenols	KH Samples
1	0.619	C_20_H_18_O_12_	Xylopyranosyl quercetin	EH, RH
2	0.633	C_15_H_14_O_6_	Catechin	BH, RH
3	0.638	C_9_H_10_O_5_	Syringic acid	RH
4	0.658	C_15_H_18_O_9_	Caffeoyl-D-glucose	BH, RH
5	0.662	C_30_H_22_O_19_	Quercetin glucoside	All
6	0.673	C_9_H_8_O_4_	Caffeic acid	RH, AH
7	0.686	C_7_H_12_O_6_	Quinic acid	RH
8	0.689	C_6_H_6_O_3_	Phloroglucinol	BH
9	0.69	C_25_H_24_O_12_	3,5-Di-*O*-caffeoylquinic acid	BH, RH
10	0.705	C_15_H_10_O_6_	Kaempferol	BH
11	0.721	C_10_H_10_O_4_	Ferulic acid	RH
12	0.722	C_28_H_32_O_19_S	Quercetin 3'-methyl ether 3-(4''-sulfatorutinoside)	AH, EH, RH
13	0.83	C_15_H_12_O_5_	Naringenin	RH
14	0.834	C_22_H_18_CO_12_	Chicoric acid	RH
15	0.949	C_9_H_8_O_3_	Hydroxycinnamic acid	RH
16	1.711	C_15_H_10_O_8_	Myricetin	RH

**Table 4:** Type of identified polyphenols and their relative quantification in Kelulut honey

Polyphenols	Relative Quantification (a.u.)			
	**AH**	**BH**	**EH**	**RH**
3,5-Di-*O*-caffeoylquinic acid	-	1.04	-	1.26
Caffeic acid	0.79	-	-	5.92
Caffeoyl-D-glucose	-	13.51	-	13.69
Catechin	-	1.37	-	2.44
Chicoric acid	-	-	-	1.23
Ferulic acid	-	-	-	1.66
Hydroxycinnamic acid	-	-	1.77	0.92
Kaempferol	-	2.16	-	-
Myricetin	-	-	-	1.00
Naringenin	-	-	-	2.34
Phloroglucinol	-	0.32	-	-
Quercetin 3'-methyl ether 3-(4''-sulfatorutinoside)	2.11	-	1.01	0.59
Quercetin glucoside	0.69	1.94	1.69	1.52
Quinic acid	-	-	-	0.61
Syringic acid	-	-	-	4.55
Xylopyranosyl quercetin	-	-	6.40	8.09

- indicates not detected

### In silico approach for the prediction of the mechanism of inhibition for KH

As shown in Supplementary Table 1, the amino acid residues that potentially acted as active interaction sites for the signalling protein molecules were identified based on SPPIDER II. In general, the signalling protein molecules were selected based on their key roles in PMACI-induced mast cell degranulation. Calmodulin-1 and PKC alpha were selected as they are the upstream proteins related to the influx of calcium ions during mast cell degranulation [29]. MAPKs (p38α, ERK1, and JNK1) and NF-κB are the downstream signalling proteins that play important roles during mast cell activation, which eventually leads to the release of inflammatory mediators [30]. The total number of active interaction sites for p38α, ERK1, JNK1, NF-κB, Calmodulin-1, and PKC alpha were 116, 39, 51, 27, 57, and 65 respectively.

To proceed with the molecular docking, the two potent KHs—RH and BH, were selected. Each of the signalling proteins molecules was subjected to protein–ligand docking using the HADDOCK server. The HADDOCK scores are shown in Table 5. Generally, the docking between p38, NF-κB, Calmodulin-1, and PKC alpha with quercetin glucoside compound were the best docking complexes (lowest HADDOCK score) among all the polyphenols whereas the polyphenol with the best binding between ERK and JNK was 3,5-di-*O*-caffeoylquinic acid. The HADDOCK scores of these six best docked complexes ranged from -7.5 to 43.0 a.u. As a lower HADDOCK score indicated a more favourable binding, the docking between 3,5-di-*O*-caffeoylquinic acid and ERK was the most favourable because it had the lowest HADDOCK score of -7.5 ± 2.5 a.u. It was followed by p38α with the quercetin glucoside compound (-5.1 ± 5.6 a.u), Calmodulin-1 with the quercetin glucoside compound (13.3 ± 9.1 a.u), JNK with 3,5-di-*O*-caffeoylquinic acid (15.3 ± 6.7 a.u), NF-κB with the quercetin glucoside compound (22.0 ± 9.6 a.u), and lastly, PKCα with the same quercetin glucoside compound (43.0 ± 8.9 a.u).

**Table 5:** HADDOCK score between identified polyphenols by LC–MS/MS with selected signalling protein molecules

Polyphenol	HADDOCK score (a.u.)					
	p38α	JNK	ERK	NF-κB	PKCα	Calmodulin-1
3,5-Di-*O*-caffeoylquinic acid	9.6 ± 2.8	15.3 ± 6.7	-7.5 ± 2.5	25.7 ± 4.6	44.9 ± 5.1	23.8 ± 0.9
Caffeic acid	28.7 ± 6.4	37.7 ± 3.8	27.9 ± 3.0	41.7 ± 9.3	84.0 ± 5.2	59.7 ± 1.9
Caffeoyl-D-glucose	18.1 ± 2.2	29.2 ± 3.3	23.3 ± 1.3	34.3 ± 8.9	68.6 ± 9.1	41.2 ± 3.5
Catechin	18.1 ± 7.2	32.9 ± 1.2	25.5 ± 6.0	41.5 ± 2.9	73.1 ± 7.7	47.3 ± 4.7
Chicoric acid	1.7 ± 5.9	23.9 ± 7.2	2.9 ± 7.5	23.5 ± 14.6	58.6 ± 5.5	30.3 ± 10.5
Ferulic acid	24.4 ± 5.8	39.8 ± 4.4	31.8 ± 7.2	39.9 ± 5.3	98.1 ± 0.9	59.7 ± 3.5
Hydroxycinnamic acid	18.3 ± 1.0	43.7 ± 4.6	26.2 ± 8.2	42.4 ± 11.7	93.9 ± 12.7	60.9 ± 5.1
Kaempferol	18.0 ± 2.0	65.2 ± 4.9	46.0 ± 4.7	58.7 ± 12.7	99.3 ± 10.0	65.1 ± 4.7
Myricetin	6.0 ± 1.7	29.7 ± 3.1	22.4 ± 7.0	33.2 ± 5.6	73.6 ± 9.1	41.8 ± 4.7
Naringenin	-0.5 ± 9.7	34.0 ± 5.4	26.1 ± 5.4	45.8 ± 6.9	77.6 ± 19.3	44.1 ± 5.9
Phloroglucinol	38.6 ± 4.3	63.4 ± 4.0	42.9 ± 2.2	47.0 ± 13.6	96.3 ± 11.2	66.9 ± 7.0
Quercetin 3'-methyl ether 3-(4''-sulfatorutinoside)	16.7 ± 14.6	30.6 ± 2.5	2.8 ± 3.0	27.0 ± 6.8	55.9 ± 8.5	36.6 ± 7.4
Quercetin glucoside	-5.1 ± 5.6	24.7 ± 4.7	0.2 ± 2.6	22.0 ± 9.6	43.0 ± 8.9	13.3 ± 9.1
Quinic acid	26.2 ± 4.7	54.0 ± 5.5	27.6 ± 5.2	47.7 ± 3.3	93.5 ± 5.0	64.0 ± 4.2
Syringic acid	24.6 ± 5.8	50.9 ± 10.7	35.7 ± 8.7	45.4 ± 3.2	90.7 ± 21.6	66.2 ± 11.3
Xylopyranosyl quercetin	2.8 ± 4.3	21.3 ± 5.1	11.3 ± 2.3	29.3 ± 5.1	53.5 ± 12.8	26.7 ± 5.0

The six signalling protein molecules-polyphenol docked complexes proceeded for the binding affinity prediction using the PRODIGY server to determine their binding affinity. Although the high-abundant polyphenol in BH and RH, namely caffeoyl-D-glucose was not ranked as the best-docked complexes, the compound was still undergone with PRODIGY analysis to determine the binding affinity. The results for predicted binding energy in terms of ΔG from each docked complexes are shown in Table 6. In general, the ΔG of the docked complexes showed range of value between -6.1 to -9.5 kcal/mol. The highest ΔG value was the complex formed between 3,5-di-*O*-caffeoylquinic acid with JNK (-9.5 ± 0.06 kcal/mol), whereas the complex formed between caffeoyl-D-glucose and NF-κB has the lowest energy release (-6.1 ± 0.06 kcal/mol) among the selected docked complexes. Hence, the interaction of former complex is the least favourable among all whereas the latter complex is more favourable based on the binding affinity.

**Table 6:** PRODIGY score and predicted inhibition constant value for docked complexes

Polyphenol	PRODIGY score, ΔG in Kcal/mol (Predicted K_i_, µM)					
	p38α	JNK	ERK	NF-κB	PKCα	Calmodulin-1
Quercetin glucoside	-9.2 ± 0.14 (0.18)			-8.4 ± 0.13 (0.69)	-8.7 ± 0.30 (0.41)	-8.8 ± 0.30 (0.35)
3,5-Di-O-caffeoylquinic acid		-9.5 ± 0.06 (0.11)	-9.3 ± 0.08 (0.15)			
Caffeoyl-D-glucose	-7.4 ± 0.06 (3.71)	-7.6 ± 0.13 (2.65)	-7.4 ± 0.05 (3.71)	-6.1 ± 0.06 (33.4)	-7.7 ± 0.10 (2.24)	-7.6 ± 0.01 (2.65)

**Fig. 8:**
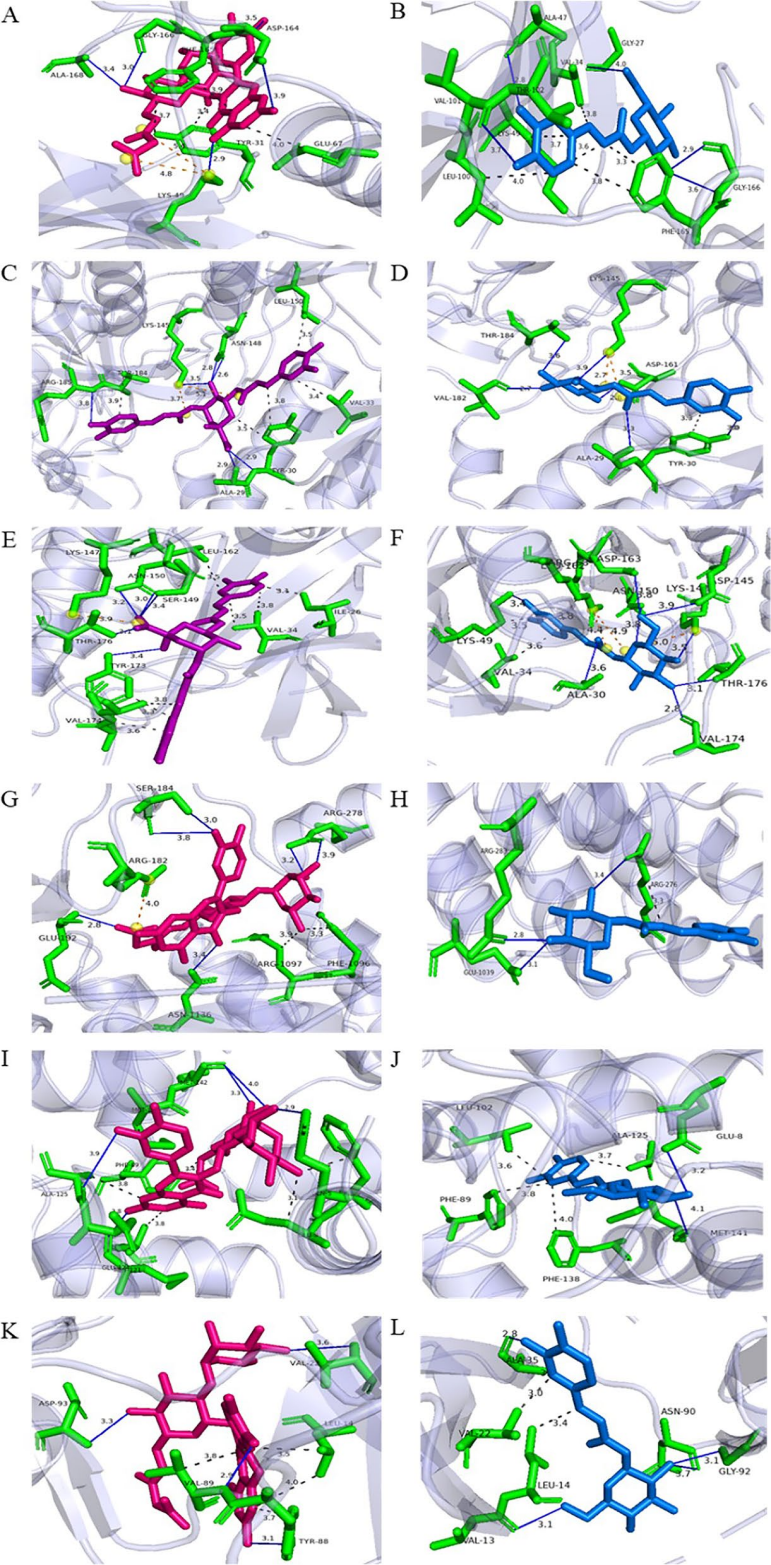
The in silico interactions between selected signalling protein molecules and polyphenol ligands. **A** and **B** p38α complex, (**C** and **D**) ERK complex, (**E** and **F**) JNK complex, (**G** and **H**) NF-κB complex, (**I** and **J**) Calmodulin-1 complex, (**K** and **L**) PKCα complex. Amino acid residues involved in interaction were shown in green, quercetin glucoside ligand was shown as pink structure, 3,5-di-*O*-caffeoylquinic acid was shown as purple structure and caffeoyl-D-glucose ligand was shown as blue structure. Hydrogen bonds were represented by blue lines, and hydrophobic interactions were represented by grey dotted lines

Protein–Ligand Interaction Profiler (PLIP) was used to identify the types of protein–ligand interaction within a complex. The interactions, which included the key amino acid residues within the signalling protein molecules that interacted with the three selected polyphenols (caffeoyl-D-glucose, 3,5-di-*O*-caffeoylquinic acid, and quercetin glucoside compound) were shown in Fig. 8, whereas the types and number of interactions were listed in Supplementary Table 2. In general, the types of interactions that can be identified in all the docked complexes were hydrophobic (1 to 7 interactions), hydrogen bonds (1 to 6 interactions), and salt bridges (0 to 2 interactions) between the signalling protein molecules and polyphenol ligands.

According to Supplementary Table 2, it was shown that 3,5-di-*O*-caffeoylquinic acid, the quercetin glucoside compound, and caffeoyl-D-glucose were having the most interactions between JNK, p38α, and PKCα, with a total of 13, 12 and 10 interactions respectively. There were seven hydrophobic interactions, five hydrogen bonds, and one salt bridge predicted for the JNK with a distance ranging from 2.9 to 3.9 Å. Whereas for p38α, there were five hydrophobic interactions, five hydrogen bonds, and two salt bridges with a distance ranging from 2.9 to 5.4 Å. Finally, the PKCα was predicted to contain four hydrophobic interactions and six hydrogen bonds within a 2.8 to 3.7 Å distance. For ERK signalling protein molecule, it was predicted to interact with 3,5-di-*O*-caffeoylquinic acid through four hydrophobic interactions, six hydrogen bonds, and two salt bridges (2.09 to 5.08 Å). Whereas for NF-κB and Calmodulin-1, both of which were predicted to interact more readily with the quercetin glucoside compound.

## Discussion

KH is a local stingless bee honey from Trigona bees found in Malaysia that has been used as a remedy for colds, coughs, and respiratory illnesses [31]. The bioactivity of KH is believed to rely on the polyphenol contents in the honey as studies have reported that this honey has a rich content of polyphenols including phenolic compounds and flavonoids when compared to other kinds of honey [32, 33]. In recent years, KH has been reported for its anti-inflammatory and antioxidant effects in both in vitro and in vivo models through the suppression of inflammatory cytokines and inducible nitric oxide synthase [34, 35]. However, there are no studies that have yet to highlight the anti-allergic effects of KH through MC degranulation since MC activation is a key event in allergic diseases [36]. According to a previous reported study, another type of honey such as Tualang honey has shown promising results in elevating the symptoms of allergic rhinitis [37]. As KH contains a higher level of bioactive compounds that may potentially exhibit superior anti-allergy effects, thus the present study aimed to investigate the inhibitory effects of KH against allergic diseases by using an in vitro model of PMACI-induced HMC cells.

The release of allergic mediators due to MC activation is reliant on the influx of calcium ions into the cell, which is achievable by the use of PMACI in the present study [7]. These mediators such as histamine and β-hexosaminidase are the reason behind the manifestation of allergic symptoms such as increased vascular permeability, constriction of smooth muscle, secretion of mucus, itchiness, and pain sensation [38]. In this study, three non-cytotoxic concentrations of KHs, as determined by the CCK-8 cell viability assay, were used in subsequent MC mediators release assays. The current findings showed that the histamine and β-hexosaminidase levels were significantly increased with PMACI induction. However, these two mediators’ levels were significantly suppressed concentration-dependently in two of the KH pre-treatment, which were BH and RH. This result was somewhat similar to a previous reported study demonstrating the anti-allergic properties of Manuka honey in terms of lowering the histamine level [39]. However, such inhibitory effects were not observed in another two KHs (AH and EH), showing that KHs from different botanical sources may affect MC degranulation differently. The difference in bioactivity due to botanical sources has been documented in other studies. For example, Ranneh et al. (2018) has demonstrated that the total antioxidant activity of honey from a monofloral source was different from honey harvested by bees from a multifloral source in both KH and Tualang honey [40]. This finding was similar to another study reporting the effect of botanical sources on the antioxidant, antimicrobial, and anti-inflammatory properties of *Apis* honey [20]. Interestingly, another study done by Gül & Pehlivan (2018) suggested that the geographical locations of the bee hive would affect the antioxidant properties of honey [41]. However, the geographical factor is not contributing to the different responses of KH in this study because all the KH samples were collected from the same bee farm. Collectively, these have proven that the bioactivities of honey are more dependent on the phytochemical content of the harvested honey. Therefore, it is important to determine the physiochemical profile of the honey to gain a full understanding of the anti-allergic properties of KH against activated MC.

Apart from the release of preformed mediators, the release of de novo synthesized inflammatory mediators such as TNF-α, IL-6, IL-8, and IL-4 by activated MC in a prolonged allergic reaction was well-reported in previous studies [36, 38]. As these mediators contribute to the pathogenesis of allergic diseases through the activation and recruitment of other immune cells, as well as the manifestation of allergic symptoms, the reduction of inflammatory mediator levels is important for the alleviation of an inflammatory response [42–45]. Hence, the release of inflammatory mediators was evaluated in this study. From the ELISA results, AH and EH showed no significant effect on the inhibition of inflammatory mediators. However, it was shown again that BH and RH exhibited inhibitory effects on the release of TNF-α, IL-6, IL-8, and IL-4 significantly at higher concentrations (0.5 and 1%), suggesting that they were more potent as compared to the former two KHs. The inhibitory effects of BH and RH may relate to previous reported studies where aqueous extract (2 to10 mg/kg) from the root bark of rubber tree and alcoholic extracts (50 and 100 µg/mL) of bamboo leaves have been shown to contain potent anti-inflammatory effects using an in vivo and in vitro model respectively [46, 47]. Moreover, such an inhibitory effect by honey was in line with a study conducted by Kim et al., (2018), whereby the production of IL-6 was inhibited in LPS-induced RAW264.7 cells with 50–200 µg/mL of acacia honey [48]. Ranneh et al. (2019) also reported that 4.6 g honey/kg and 9.3 g honey/kg of KH successfully attenuated inflammation by reducing the levels of inflammatory mediators including TNF-α, IL-6, and IL-8 in LPS-induced rats [35].

In contrast, another study demonstrated that the release of IL-4 was decreased in 3% of Manuka honey but the same concentration of honey increased the production of TNF-α in a neutrophil cell line (differentiated HL-60 cell) [49]. However, Tonks et al. (2003) reported that an increase in the secretion of inflammatory mediators (TNF-α, IL-1β, and IL-6) was observed in both human peripheral blood monocytes cells and precursors of macrophages (MM6) cells when treated with 1% of honey after a 24 h incubation as compared to normal cells [50]. The results from these two studies were contradicting the current findings which are possibly due to the difference in honey and inducer used in the studies. The inducer used in the studies by Tonks et al. (2003) and Minden-birkenmaier et al. (2019) was LPS, a major component of the gram-negative bacteria cell wall to trigger an inflammatory response as a key pathogenic stimulator [49–51]. It might also be due to the presence of a small number of allergens in the raw Manuka honey that triggers the inflammatory response [52].

The differences in anti-inflammatory effects among the KHs were reflected in the release of mediators, it has sparked the interest to understand whether the differences in phytochemical profile, particularly polyphenol contents play a part in their anti-allergic effects. In this study, it was found that the TPCs of KH were quite high, especially in the potent KHs which are BH and RH. The result aligned with other studies that claimed the polyphenol content of honey is directly associated with its bioactivities [40, 53, 54]. Whereby, KHs with higher TPC, namely BH and RH samples possessed significant anti-inflammatory activities by reducing the inflammatory mediators’ release as compared to AH and EH. In comparison with the TPC of KH from other studies, it was shown that BH has a similar TPC with another KH sample (1058.8 ± 2.1 mg GAE/kg honey) harvested from forest mangroves (*A. magium*) trees and flowers, but still much lower than the TPC in RH [33]. On top of that, TPC of BH and RH are five-fold of KH samples harvested from both monofloral (*A. magium*) and multifloral sources (*A. mangium, N. lappaceum, D. longan,* and *A. carambola*) ranging from 228.09 to 235.28 mg GAE/kg honey [40]. The big difference in TPC amount between the current findings with those reported by Ranneh et al., (2018) is possibly due to the use of Na_2_CO_3_ in the TPC assay from the present study [40]. The presence of Na_2_CO_3_ in alkaline conditions facilitates the reaction between polyphenols and Folin-Ciocalteu reagent [55]. Notably, even though AH and EH were shown to be less effective in regulating the inflammatory mediators' release, the TPC in these two KHs was still higher than the ones reported by Ranneh et al. (2018) [40].

The polyphenol profiles of the KH samples were evaluated using LC–MS/MS analysis to identify the polyphenols present in the samples based on our standard library information. It was found that most of the polyphenols identified belonged to phenolic acids and flavonoids which have been highlighted in their bioactivities [56–59]. Particularly, the quercetin glucoside compound was the only polyphenol found in all four KHs. This compound is a metabolite derived from quercetin. It usually acts as a pigment to give colours to fruits and vegetables. After absorption by the human body, quercetin is metabolized into different forms, for example, as quercetin glucuronide or sulfate which can be found in human plasma [60]. Based on a study by Liao & Lin (2020), 20 and 50 µM of quercetin-3-*O*-β-D-glucuronide possessed anti-inflammatory effects by modulating the ratio of pro-/anti-inflammatory (TNF-α/IL-10) cytokine gene expression in macrophages from LPS-induced murine model [61]. Besides, 3,5-di-*O*-caffeoylquinic acid was another polyphenol that was present in both BH and RH. 3,5-di-*O*-caffeoylquinic acid is a polyphenol that can be found in coffee and the di-*O*-caffeoylquinic acid family has demonstrated antioxidant and cytoprotective effects [62]. Meanwhile, 3,5-di-*O*-caffeoylquinic acid was reported to show an anti-inflammatory effect in the carrageenan-induced rat paw edema model through the suppression of TNF-α and IL-1β dose-dependently [63]. Apart from that, there was one highly abundant polyphenol presented in BH and RH, namely caffeoyl-D-glucose. Caffeoyl-D-glucose is the precursor for chlorogenic acid which is an active secondary metabolite in Japanese honeysuckle that has an anti-inflammatory and antioxidative effect [64]. Collectively, some of the identified polyphenol compounds in the KHs that have been reported to contain anti-inflammatory effects may work in synergy to inhibit the inflammatory mediators’ release during PMACI-induced MC activation. However, more future experiments will need to be done to prove this speculation.

MAPK and NF-κB signalling pathways play a crucial role in PMACI-induced MC activation and lead to the release of inflammatory mediators in an allergic reaction [30]. Previously, many studies have documented that the anti-inflammatory properties in terms of inflammatory mediators’ release are correlated with the suppression of MAPK and NF-κB signalling pathways activation through wet laboratory experiments [44, 65, 66]. As such, in the present study, an in silico approach was employed to predict the interactions between polyphenols and signalling protein molecules involved in these two pathways. Molecular docking was performed with the selected signalling protein molecules involved in the PMACI-induced MC activation against the polyphenols identified in KHs using HADDOCK server [24, 25]. Based on the results from molecular docking, it was shown that all polyphenols were able to interact with the selected signalling molecules (p38α, ERK, JNK, NF-κB, Calmodulin-1, and PKCα) through their predicted interaction sites. Among these ligands, two polyphenols i.e. the quercetin glucoside compound and 3,5-di-*O*-caffeoylquinic acid were having the best HADDOCK score based on molecular docking and highest binding affinity based on PRODIGY prediction. The in silico results were aligned with the lowered level inflammatory mediators release with BH and RH which consisted quercetin glucoside compound and 3,5-di-*O*-caffeoylquinic acid. On top of that, the latter polyphenol only presented in BH and RH. This possibly makes them one of the potential polyphenols in the BH and RH to exert their inhibitory effects on cytokines level and the inflammatory pathways.

Although the docked complexes between signalling molecules and the abundant polyphenol (caffeoyl-D-glucose) were included for binding affinity analysis, the ΔG of these complexes were generally much higher as compared with those that interacted with the quercetin glucoside compound or 3,5-di-*O*-caffeoylquinic acid. The current computational findings suggest that caffeoyl-D-glucose was predicted to potentially binds to MAPKs, NF-κB, Calmodulin-1, and PKCα, but the interactions were not as stable as the quercetin glucoside compound and 3,5-di-*O*-caffeoylquinic acid. The binding energy results showed the interactions with downstream MAPKs seem to be possibly more stable than the other three upstream signalling molecules. Nevertheless, all docked complexes between MAPKs and the three selected polyphenols had interacted through hydrophobic interactions, hydrogen bonds, and salt bridges.

**Fig. 9:**
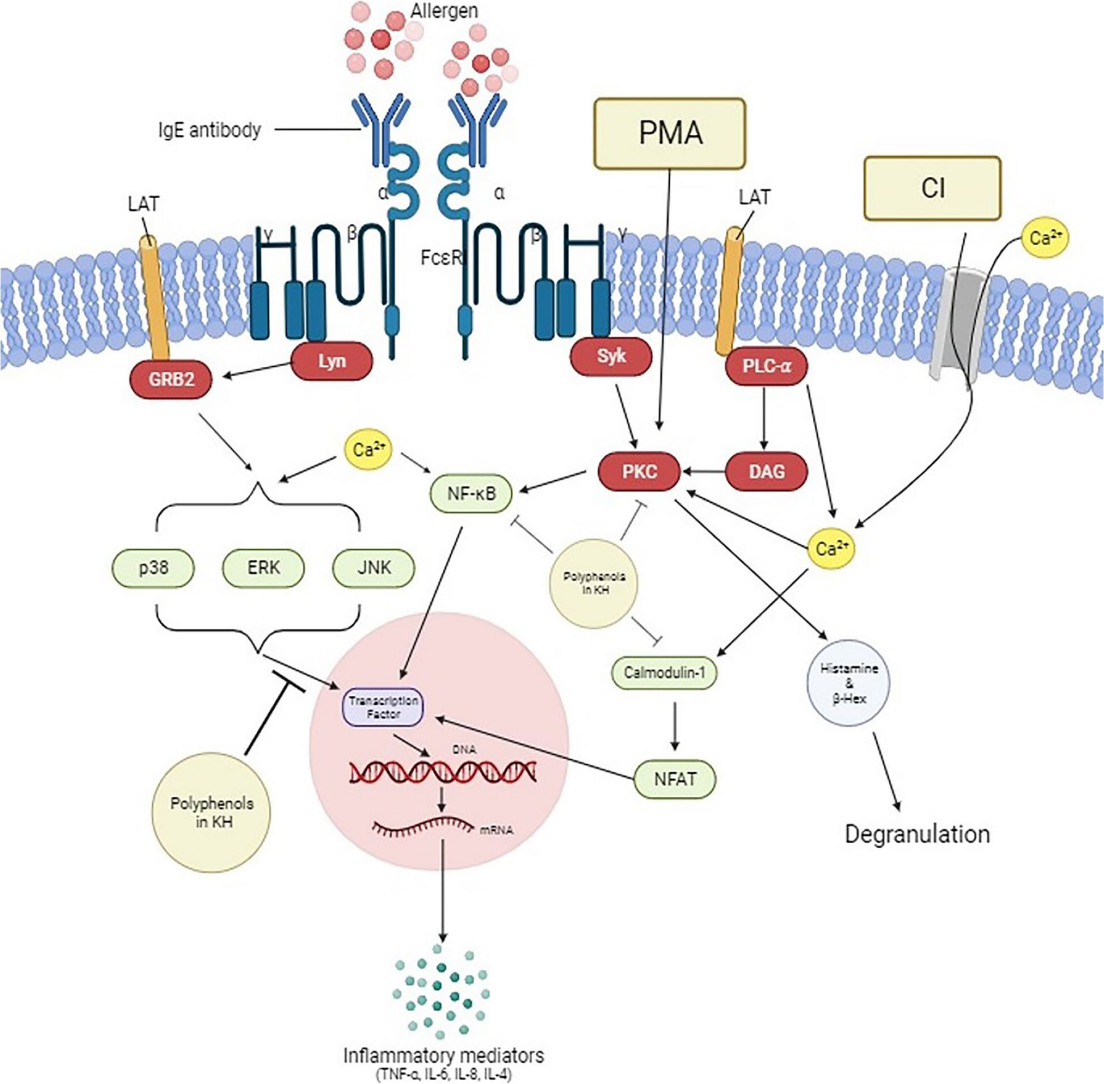
The graphical illustration of the inhibitory effects of KH in a PMACI-induced mast cell activation. Apart from the IgE-mediated mast cell activation, a mast cell can be stimulated to release allergic-related inflammatory mediators, such as histamine, β-hexosaminidase, IL-4, IL-6, IL-8, and TNF-α by the use of PMACI. An increase in intracellular calcium level due to PMACI causes activation of the signalling pathways associated with mast cell stimulation. The present study demonstrated that polyphenols from stingless bee honey were able to inhibit the activation of mast cells by interacting with some of the signalling molecules such as MAPKs (p38α, ERK, and JNK), NF-κB, Calmodulin-1, and PKC alpha. In particular, the interaction of the polyphenols with MAPKs is stronger than the other 3 signalling molecules (denoted by a larger inhibition sign)

The presence of hydrophobic interactions can be associated with binding affinity whereby the binding affinity increases between the protein–ligand interfaces when there is hydrophobic interaction. On the other hand, hydrogen bonds play a key role in ligand recognition, giving the most stabilizing forces in a biological system [67]. In addition, salt bridge strength is much stronger than typical hydrogen bonding [68]. Therefore, their presence between the binding polyphenols with MAPKs further strengthens the interaction. Additionally, 3,5-di-*O*-caffeoylquinic acid and caffeoyl-D-glucose only presented in BH and RH which had shown significant inhibitory effects on the inflammatory mediators’ release. Based on the findings, these two polyphenols may play a significant role in the anti-allergic effect of KHs through the interaction with activated MAPKs that leads to the reduction of inflammatory response. Collectively, one may deduce that the combination of PMA and CI may activate a MC to release allergic mediators through the activation of MAPKs, NF-κB, Calmodulin-1, and PKC alpha (Fig. 9). Even though all the identified phytochemicals in BH and RH are shown to interact with the selected signalling molecules to a certain extent, the quercetin glucoside compound and 3,5-di-O-caffeoylquinic acid are more likely the main compounds that play the inhibitory role within an activated MC. Specifically, the activated MAPKs are predicted to be the favorable binding targets of the identified polyphenols in BH and RH, resulting in the inhibition of the MAPKs activity and subsequence suppression of the inflammatory mediators’ production. In addition, speculation can also be made where all the potent polyphenols are able to work synergistically to exert the overall inhibitory effects of the KHs against PMACI-activated MC. However, the results from docking and interaction between the proteins and polyphenols ligand were a prediction based on computational calculations, the potential inhibitory effects of the polyphenols will still need to be validated through future experiments. To the best of our knowledge, there are yet to be any wet lab experiments from reported studies that indicate the interaction between these two polyphenols with the MAPKs.

## Conclusion

In conclusion, the present study exhibited that KH contains potential anti-allergic effects. However, the KH from different botanical sources showed different anti-allergic potentials in terms of inhibition of MC degranulation and inflammatory mediators’ release. Particularly, BH and RH that are rich in polyphenol content, such as the 3,5-di-O-caffeoylquinic acid, quercetin glucoside compound, and caffeoyl-D-glucose, showed significant anti-allergic effects possibly via the interaction with activated MAPKs based on molecular docking simulation. However, more in-depth studies must be done to further justify the findings from the computational section of this study. Even though the present study demonstrated the in vitro anti-allergic effects of KH, the limitations of the in vitro model design of an experiment such as inability to fully replicate the conditions of cells in living organisms will prompt further investigations to be carried out. For example, using suitable allergy-related animal models (atopic dermatitis or asthma) and clinical trials to validate the therapeutic potential of KH in humans can be done in the future. In addition, another limitation in this study is that the KH samples were only collected from one location. This, future studies can be done on the anti-allergic effects of KH, specifically the RH and BH in this case, from other locations to determine whether geographical location may play a factor on the beneficial effects of KH. Nevertheless, the outcome of the present study has provided a preliminary scientific evidence that proves KH from specific botanical sources may contain potent polyphenols which can be developed into a potential complementary medicine for allergies in the future.

### Supplementary Information

**Additional file 1: Supplementary Table 1.** Predicted active interaction sites of the selected signalling protein molecules based on SPPIDER II. **Supplementary Table 2.** The types, number of interactions, and interacting amino acid residues between selected signalling protein molecules and polyphenols.

## Data Availability

The data generated or analysed during this study are included in this manuscript and its supplementary information files.

## Abbreviations

BSA: Bovine Serum Albumin
CCK-8: Cell counting kit-8
JNK: C-Jun N-terminal kinase
CI: Calcium ionophore
DMSO: Dimethyl sulfoxide
ELISA: Enzyme-linked immunosorbent assay
ERK: Extracellular signal-regulated kinase
FBS: Fetal bovine serum
GAE: Gallic acid equivalent
HMC: Human mast cell
Hrs: Hours
IL: Interleukin
IMDM: Isocove’s modified dulbecco’s medium
KH: Kelulut honey
LC–MS/MS: Liquid chromatography with tandem mass spectrometry
MAPKs: Mitogen-activated protein kinases
NF-κB: Nuclear factor kappa-light-chain-enhancer of activated B cells
PKCα: Protein kinase C alpha
PMA: Phorbol 12-myristate 13-acetate
PNAG: P-nitrophenyl *N*-acetyl-β-D-glucosamine
PRODIGY: Protein binding energy prediction server
TNF-α: Tumor necrosis factor-α
TPC: Total phenolic content
