# Supplementary material for: The anti-allergic potential of stingless bee honey from different botanical sources via modulation of mast cell degranulation
Source: BMC Complement Med Ther. 2023 Sep 4;23:307. doi: 10.1186/s12906-023-04129-y (PMC10476411; doi:10.1186/s12906-023-04129-y)
Supplement: Supplementary file 1 — Additional file 1: Supplementary Table 1. Predicted active interaction sites of the selected signalling protein molecules based on SPPIDER II. Supplementary Table 2. The types, number of interactions, and interacting amino acid residues between selected signalling protein molecules and polyphenols. [file 12906_2023_4129_MOESM1_ESM.docx]

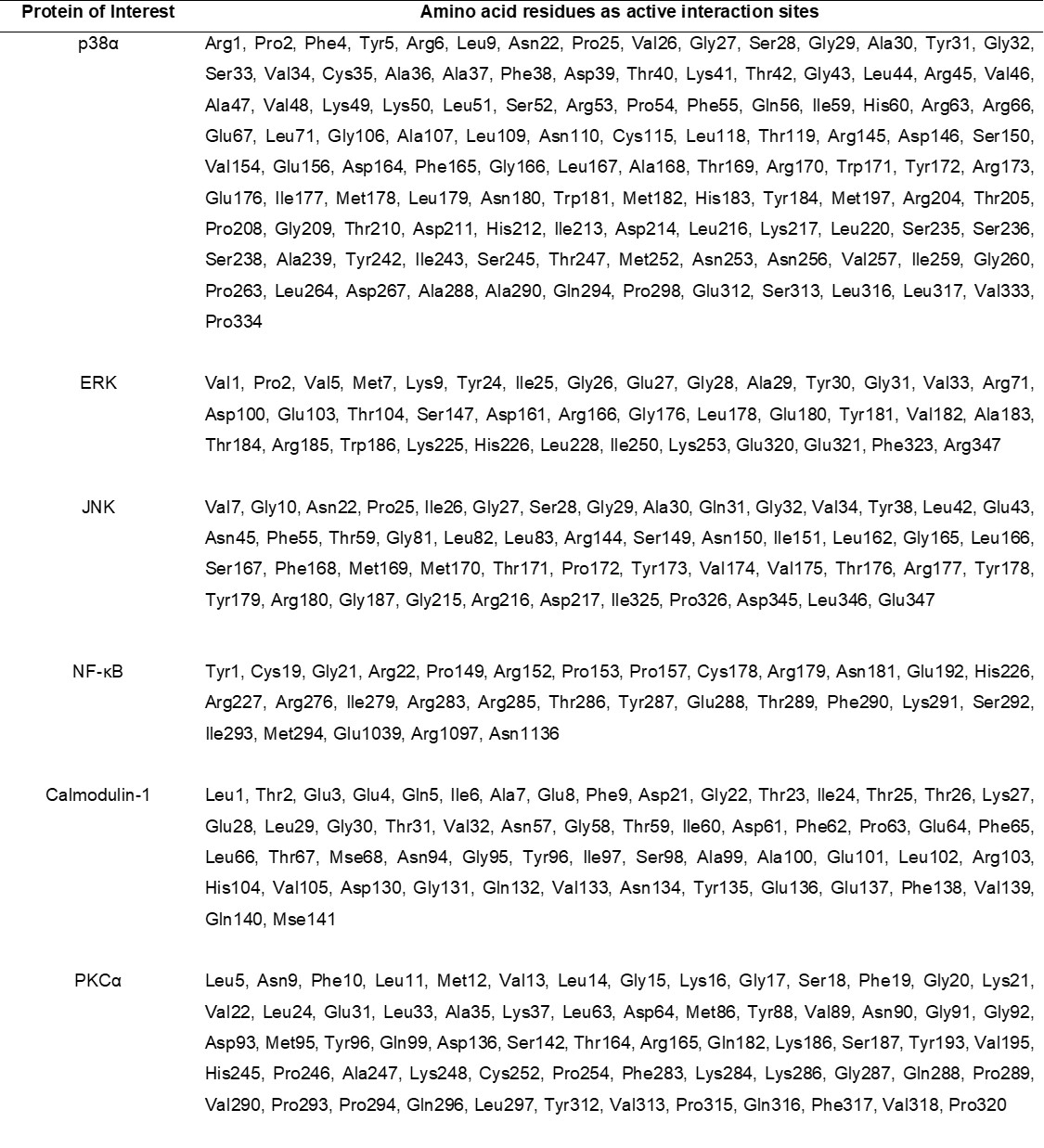
Supplementary Table 1. Predicted active interaction sites of the selected signalling protein molecules based on SPPIDER II.


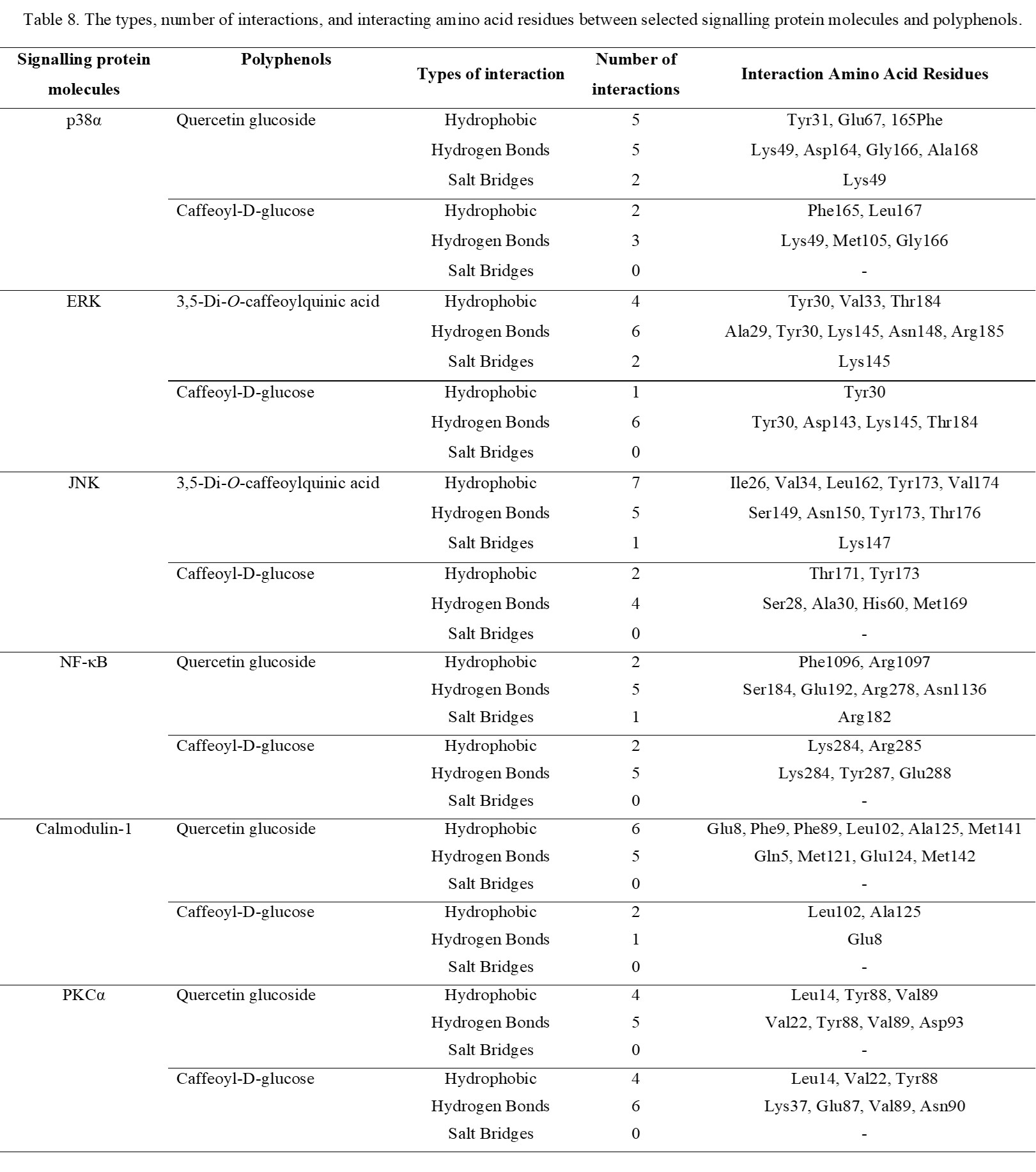
Supplementary Table 2. The types, number of interactions, and interacting amino acid residues between selected signalling protein molecules and polyphenols.
